# The role of Dodola Community Conservation Area for large mammal conservation, Ethiopia

**DOI:** 10.1186/s13104-023-06655-x

**Published:** 2023-12-20

**Authors:** Geremew Mekonnen, Zerihun Girma

**Affiliations:** 1 Farm Africa, Bale Ecoregion, Forest for Sustainable Development, Ginir, Ethiopia; 2 Department of Wildlife and Protected Area Management, Hawassa University, Hawassa, Ethiopia

**Keywords:** Conservation, Erica-scrubland, Frequency of sightings, Habitat, Sample, Species richness

## Abstract

The role of community conservation areas for large mammals is rarely evaluated. We investigated the species richness and frequency of sightings of large mammals in the Dodola Community Conservation Area. The study area was stratified into three habitat types, and 49 lines transect was laid (27 Dry evergreen Afromontane forests, 20 Sub-afro-alpine habitats, and 2 plantation forests) based on the topography, land use, and vegetation cover of the study area. A total of 24 species of large mammals were identified and recorded in the study area. Though the community conservation area is home to diverse species of mammals, including some endemic and endangered ones such as mountain nyala and Bale Monkey, human encroachment, agriculture, and overgrazing are prominent in the area, putting huge pressure on flora and fauna. Therefore, we recommend the participatory approach be strengthened to ensure sustainable coexistence between people and wildlife.

## Introduction

There has been a paradigm shift from the classical approach to wildlife conservation, which tends to fence off wildlife conservation areas from human intervention without the active participation of the local community, to a modern approach that urges the active participation of the local community right from planning to the implementation of conservation management plans. Participatory wildlife conservation has emerged over the last four decades as the most promising approach for sustainable wildlife [1–3]. The participatory approach emerged in response to the failures of top-down approaches to deliver sustainable conservation [4, 5], the local community livelihood needs, and an increasing predominance of ethical arguments fostering equality and democracy in development issues [2, 6].

Participatory Wildlife Conservation (PWM) was introduced as one of the solutions to balance conservation interests with local community livelihoods [4, 7, 8]. The core idea of PWM is to empower local community members and their traditional institutions for the sustainable conservation of wildlife resources. Through participatory wildlife conservation, the local community will gain economic benefits through ventures such as tourism and regulated wildlife harvesting [9, 10]. Experiences from south and eastern African countries revealed that community-based conservation schemes have allowed the local community to have control over wildlife and share benefits derived from the resource [3, 4]. The focus of PWM is to enhance the livelihood of the poor and often marginalized communities around wildlife conservation areas [11]. The PWM is based on the assumption that it is possible to improve rural livelihoods, conserve the environment, and promote economic growth [12]. However, detailed analyses combining socio-economic and ecological data on forest wildlife hunting are few and it is debatable if such systems can serve both economic and ecological purposes under current conditions. Although community-based conservation areas have been reported to support diverse wildlife species [13, 14], long-term wildlife monitoring data are scant.

According to [15], large mammals are those with a body weight greater than 7 kg. Ethiopia is among the most biodiversity-rich countries in Africa; it possesses over 320 species of mammals, of which 39 are endemic [16].

The Ethiopian wildlife conservation approach has been predominantly a top-down approach in that wildlife conservation is exclusively carried out by wildlife authorities with little or no participation of the local communities [14, 17, 18]. This has led to the development of a negative attitude towards wildlife resource conservation, which in turn has caused wildlife population decline and habitat degradation, fragmentation, and loss [19–21]. In Ethiopia, in response to this challenge, over the last 15 years, there has been a paradigm shift from a traditional top-down approach to a more participatory bottom-up approach that encourages local communities to actively participate in wildlife conservation decisions and considers them an important partner for conservation [8]. As a result, there have been attempts to establish community-based wildlife conservation areas in Ethiopia [22]. Among these, Menz Guassa (as old as 400 years, but interrupted from 1970 to 1991 and resumed in the late 1990s) and Adaba-Dodola Community Conservation Areas (established in 2000) are the most exemplary ones [8, 22, 23].

However, the role of these community conservation areas in wildlife conservation has rarely been evaluated. While some studies explored the socio-economic and forest conservation benefits of participatory environmental conservation for the local community as compared to the traditional top-down conservation approach [24], studies that investigated the role of community conservation areas for wildlife species conservation are scant.

Dodola community conservation is a participatory natural resource management approach whereby people, wild animals, and livestock live in harmony in the forest ecosystem nexus for sustainable natural resource management, ultimately contributing to sustainable livelihood development [22, 23]. However, it is not clear that the community conservation area contributed to the sustainable conservation of wildlife as compared to the previous traditional top-down conservation approach. As a result, the study attempts to answer the following research questions; (1) What is the large mammal species richness in the community conservation area? (2) What is the distribution of large mammal frequency of sightings along dominant habitat types in the community conservation area? (3) What is the population structure of large mammals in the community conservation area? Data on large mammal species richness, frequency of sightings and distribution is an important indicator to clearly understand the role of community conservation areas for the sustainable conservation of wildlife species. The study will also serve as baseline information for monitoring the conservation value of the area for large mammals and experiences can be extrapolated elsewhere to similar community conservation areas. Therefore, the study is aimed at investigating the role of the community conservation area for large mammal diversity and future large mammal population conservation prospects.

## Results

### Species composition and richness

A total of 24 species of large mammals grouped into 5 orders and 12 families were identified and recorded from the Dodola Community Conservation Area. The large mammal species recorded were: *Tragelaphus* *buxtoni*, *Tragelaphus* *scriptus* *meneliki*, *Redunca* *redunca*, *Sylvicapra* *grimmia*, *Oreotragus* *oreotragus,* *Hylochoerus* *meinertzhageni*, *Phacochoerus* *africanus*, *Potamochoerus* *porcus,* *Panthera* *pardus,* *Felis* *serval,* *Panthera* *pardus,* *Canis* *simensis,* *Canis* *mesomelas,* *Canis* *aureus,* *Civettictis* *civetta,* *Ichneumia* *albicauda,* *Crocuta* *crocuta,* *Mellivora* *capensis,* *Chlorocebus* *aethiops,* *Colobus* *guereza,* *Papio* *anubis,* *Chlorocebus* *djamdjamensis,* *Orycteropus* *afer* and *Hystrix* *cristata*.

The most frequently sighted large mammal species were *Sylvicapra* *grimmia,* *Phacochoerus* *africanus,* and *Papio* *anubis.* On the other hand*,* *Panthera* *pardus*, *Canis* *simensis* were among the rarely sighted mammalian species in the area. Most of the large mammalian species recorded fall under the IUCN category of Least Concern 17(71%), whereas 5(21%), were endangered and 2 (8%) were vulnerable species.

**Fig. 1:**
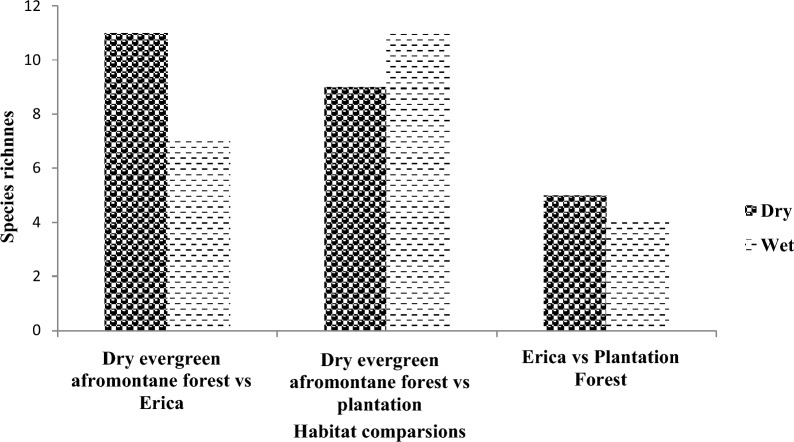
Species richness among the three dominant habitat types during dry and wet seasons

Out of a total species of large mammals recorded currently in the area, 23 species were observed during the dry season while 24 species were recorded during the wet season and the black panther (melanistic leopard) was only recorded during the wet season. The highest large mammal species richness was recorded from Dry Evergreen Afromontane Forest (DEAMF) habitats during the dry (21) and wet (20) seasons respectively (Fig. 1). The lowest large mammal species richness was recorded from the Plantation Forest (9) during the dry season (Fig. 1).

The greatest overlap of large mammalian species as calculated by the Sorenson similarity index (CC) was recorded between plantations and DEAMF and during both dry (Sorenson's Index (SI) = 0.6) and wet (SI = 0.824) seasons. The lowest large mammalian species overlap was recorded between the Erica scrubland habitat and the Plantation forest during the dry season (SI = 0.435).

### Frequency of observation per sampling effort

The frequency of observation per sampling effort of large mammalian species ranged from 0.19 to 24.90% in the dry season and 0.16 to 17.96% during the wet season (Table 1). Among the 24 species of large mammals recorded, *C.* *guereza* (24.90%), *Phacochoerus* *africanus* (16.34%), and *T.* *buxtoni* (14.59%) were the most frequently sighted species during the dry season (Table 1). However, *P.* *africanus* *(*17.96%), *C.* *guereza* *(*16.64%)*,* and *Papio* *anubis* *(*14.33%) were the most frequently observed during the wet season. Both *Canis* *simensis* and black panther (melanistic leopard) were the least frequently observed during both dry (0.19%, 0.19%) and wet seasons (0.33%, 0.16%) respectively (Table 1). When the results of both seasons are combined, *C*. *gureza* (20.43%) followed by *P.* *africanus* (17.22%) were the most sighted mammalian species in the study area, while Black panther (melanistic leopard) (0.09%) and *Canis* *simensis* (0.27%) were the least sighted species (Table 1). From 2242 individuals of large mammals recorded during the study period, 1028 (45.84%) were observed during the dry season and 1214 (54.15%) were observed during the wet season (Table 1).

**Table 1:** Large mammalian species observed and counted using visual sighting and respective rank during both dry and wet seasons in Dodola Community Conservation Area

Species type	Number of individuals Observed per given km walked			Frequency of sightings per sampling effort (%)					
	Dry/3780 km	Wet/3780 km	Over all/7560 km	Dry	Rank	Wet	Rank	Overall	Rank
*Colobus* *guereza*	256	202	458	24.9	1st	16.64	2nd	20.43	1st
*Phacochoerus* *africanus*	168	218	386	16.3	2nd	17.96	1st	17.22	2nd
*Tragelaphus* *buxtoni*	150	166	316	14.6	3rd	13.67	4th	14.09	3rd
*Papio* *anubis*	87	174	261	8.46	5 rd	14.33	3rd	11.64	4th
*Tragelaphus* *sriptus* *menelikis*	97	155	252	9.44	4th	12.77	5th	11.24	5th
*Chlorocebus* *aethiops*	49	70	119	4.77	7th	5.77	6th	5.31	6th
*Sylvicapra* *grimmia*	55	47	102	5.35	6th	3.87	7th	4.55	7th
*Oreotragus* *oreotragus*	34	42	76	3.31	8th	3.46	8th	3.39	8th
*Redunca* *redunca*	25	23	48	2.43	9th	1.89	9th	2.14	9th
*Chlorocebus* *djamdjamensis*	14	15	29	1.36	10th	1.24	10th	1.29	10th
*Potamochoerus* *porcus*	12	14	26	1.17	12th	1.15	11th	1.16	11th
*Crocuta* *crocuta*	13	9	22	1.26	11th	0.74	14th	0.98	12th
*Hystrix* *cristata*	9	12	21	0.88	14th	0.99	12th	0.94	13th
*Panthera* *pardus*	10	7	17	0.97	13th	0.58	16th	0.76	14th
*Civettectis* *civeta*	8	9	17	0.78	15th	0.74	14th	0.76	15th
*Orycteropus* *afer*	7	10	17	0.68	16th	0.82	13th	0.76	16th
*Ichneumia* *albicauda*	8	5	13	0.78	15th	0.41	18th	0.58	17th
*Canis* *aureus*	5	8	13	0.49	17th	0.66	15th	0.58	18th
*Hylochoerus* *meinertzhageni*	5	7	12	0.49	17th	0.58	16th	0.54	19th
*Felis* *serval*	4	6	10	0.39	18th	0.49	17th	0.45	20th
*Canis* *mesomelas*	4	6	10	0.39	18th	0.49	17th	0.45	21st
*Mellivora* *capensis*	4	5	9	0.39	18th	0.41	19th	0.4	22th
*Canis* *simensis*	2	4	6	0.19	19th	0.33	20th	0.27	23th
*Black* *panthera*	0	2	2	0.19	19th	0.16	21th	0.09	24th
Total	1026	1216	2242	100		100		100	

The maximum number of large mammal individuals was observed and counted in the DEAMF habitat (559) during the wet season and the lowest was recorded in the Erica habitat (185) during the dry season (Table 2).

**Table 2:** Frequency of observation and distribution of large mammals among dominant habitat types at Dodola Community Conservation Area

Family	species type	Number of animals sighted per given line transect distance walked					
		DGAMF (3240 km walked)		Erica (4000 km walked)		Pl 320 km walked	
		Dry	Wet	Dry	Wet	Dry	Wet
Bovidae	*T.* *buxtoni*	80	41	70	125	0	0
	*T.s.* *meneliki*	93	100	4	0	0	55
	*R.* *redunca*	8	7	5	0	12	16
	*S.* *grimmia*	18	14	10	9	27	24
	*O.* *oreotragus*	0	0	34	42	0	0
Suidae	*H.* *meinertzhageni*	5	7	0	0	0	0
	*Ph.* *africanus*	45	81	22	42	101	95
	*P.* *porcus*	12	14	0	0	0	0
Felidae	*P.* *pardus*	7	5	3	2	0	0
	*F.* *serval*	4	4	0	2	0	0
	*P.* *pardus(melanistic)*	0	0	0	2	0	0
canidae	*C.* *simensis*	0	0	2	4	0	0
	*C.* *mesomelas*	1	2	1	4	2	0
	*C.* *aureus*	4	0	1	2	0	6
Vivarridea	*C.* *civetta*	8	3	0	0	0	6
Herpestidae	*I.* *albicauda*	6	2	2	0	0	3
Hyaenidae	*C.* *crocuta*	3	3	1	1	9	5
Mustelidae	*M.* *capensis*	3	5	0	0	1	0
Colobidae	*Ch.* *aethiops*	15	28	0	0	34	42
	*C.* *guereza*	160	131	0	0	96	71
Cercopithecidae	*P.* *anubis*	57	84	30	0	0	90
	*Ch.* *djamdjamensis*	14	15	0	0	0	0
Orycteropodidae	*O.* *afer*	2	10	0	0	5	0
Hystricidae	*H.* *cristata*	9	3	0	0	0	9
Total no. of species per habitat		21	20	14	11	9	12
Total number of individuals observed		554	559	185	235	287	422

*DEAMF*  Dry Evergreen Afromontane forest, *ERI*  Erica scrubland, *Pl*  Plantation forest

Seasonal variation in the frequency of counting individuals of species was also observed in all species in the study area. *C*. *gureza* (160) in the DEAMF, *T*.*s*. *buxtoni* (70) in the Erica scrubland habitat, *Ph.* *africanus* (101) was the most frequently observed species in the DEAMF, Erica and plantation habitats, respectively, during the dry season. However, during the wet season, the most frequently counted species was *C*. *guereza* (131) in DEGAMF, *T*. *buxtoni* (125) in Erica scrubland, and *Ph.* *africanus* (95) in plantation habitat, respectively (Table 2).

### Population structure

*T.* *buxtoni,* *T.* *s.* *menelikis,* *C.* *gureza,* *Sylvicapra* *grimmi*a and *Chlorocebus* *djamdjammensis* had large proportions of adult age group (Male adult and Female adult) individuals, with the highest adult female individuals, during both seasons during the 2021 survey. But, the populations of *P.* *africanus* and *P.* *anubis* had a relatively higher proportion of sub-adult and young age groups as compared to other species during both seasons (Table 3). The adult male to adult female sex ratio for the 3 endemic species was as follows; the population ratio of the species *T.* *buxtoni* (1:12), *T.* *scriptus* *meneliki* (1:11), and *Ch.* *djamdjamensis* (1:21) was observed (Table 2). A relatively higher sub-adult to young ratio was also recorded for *T*. *s*. *meneliki* (Table 3).

**Table 3:** Age and sex distribution of sexually dimorphic large mammals in Dodola Community Conservation Area during the year 2021

No. of animals observed during both seasons	Number of individual species							
Name of species	Age and sex							
	Male adult	Female adult	Ratio (M:F)	Sub-adult	young	Ratio (SA:Y)	Unknown	Total
*T.* *buxtoni*	76	112	1:47	55	73	1:32	0	316
*T.* *s.* *meneliki*	76	90	1:18	48	35	1:0.73	3	252
*R.* *redunca*	3	10	1:33	5	9	1:80	21	48
*H.* *meinertzhageni*	1	1	1:1	2	2	1:1	6	12
*C.* *simensis*	0	0	0	0	0	0	6	6
*P.* *africanus*	80	105	1:31	54	117	1:2.17	30	386
*C.* *aethiops*	22	38	1:72	20	27	1:1.35	12	119
*P.* *anubis*	45	55	1:22	65	74	1:14	22	261
*C.* *guereza*	81	146	1:80	74	114	1:54	43	458
*P.* *porcus*	4	7	1:75	9	3	1:0.33	3	26
*S.* *grimmia*	40	33	1:0.82	10	12	1:1.20	7	102
*C.* *mesomelas*	0	0	0	0	0	0	10	10
*C.* *aureus*	0	0	0	0	0	0	13	13
*O.* *oreotragus*	1	1	1:1	0	13	0	61	76
*C.* *djamdjamensis*	3	7	1:1.75	3	4	1:33	4	21

*M*  male, *F* female adult

## Discussion

### Species composition and richness

The results of the large mammals' survey indicate that the community conservation area is home to diverse large mammalian species (24 species) including endemic and endangered species comparable to national parks, including the adjacent Bale Mountains National Park (BMNP) and neighboring Arsi Mountains National Park (AMNP). This could indicate that protected areas other than national parks, such as community conservation areas, are important conservation areas for wildlife species elsewhere in east Africa [10, 14, 25]. Particularly, the community conservation area harbors comparable large mammals species richness with community conservation and national parks elsewhere in Ethiopia. For instance, [14], reported 8 species of large mammal from the Humbo Community Conservation Area. Similarly, [26] recorded 21 medium and large mammals in the Jorgo-Wato protected forest, in western Ethiopia. Michole Community Protected Forest was reported to harbor 17 species of large and medium-sized mammals [19].

The heterogeneous plant species assemblage available could contribute to the high richness of mammalian species in this study [22, 23]. Several scholars [27, 28] showed a positive correlation between habitat heterogeneity and animal species richness. Furthermore, the occurrence of four endemic species (mountain nyala, Menelik’s bushbuck, bale monkey, and Ethiopian wolf) and three vulnerable species (Bale monkey, leopard and black leopard), one endangered (mountain nyala) and one critically endangered (Ethiopian wolf) species signify the great conservation value of the area. Previous floristic studies in Dodola Community Conservation Area also reported that plant species richness and abundance and structural complexity are higher in the community-managed forest than in adjacent state-managed forest and as compared to the former status (when it was managed by the state) [24]. Particularly, forest cover increased by 15.6 between 2002 and 2006, after the establishment of the community conservation area, whereas the adjacent state-managed forest cover declined by almost the same amount (16%) during the same time [24].

### Frequency of observation per sampling effort

The significant seasonal variation in large mammals observed and counted is related to food availability [25, 28] and the intensity of disturbance. During the wet season, because of abundant rainfall, there were better food, cover, and water sources than in the dry season, which increased the number of large mammals observed and counted during the wet season [29, 30]. On the other hand, they could move out of the community conservation area during the dry season in search of a better source of food. Similar Studies elsewhere in a different part of Ethiopia have revealed that species richness is often high in areas where there are sufficient foods, water, cover, and space [25, 31, 32].

The results of the study revealed that *Colobus* *guereza,* *Phacochoerus* *africanus,* *Tragelaphus* *buxtoni*, and *Papio* *anubis* were the most frequently sighted species relative to the total recorded individuals. *Phacochoerus* *africanus* is a widely distributed ungulate known to occur in almost all of Africa and sub-Saharan countries in diverse habitat types [33]. Warthogs reach sexual maturity fast (2 years) and give up to 8 piglets per litter in food-abundant areas, which makes them have a fast-growing population [34, 35]. Furthermore, warthogs graze different species of annual grasses and perennial plant materials, including human-inhabited areas, which makes them adapt and reproduce fast over different habitat types, including in areas where human disturbances are intense [33, 35]. Likewise, *Papio* *anubis* also occurs in a wide range of habitat types, including human-inhabited areas, and feeds on diverse food items [28]. In general, the conducive habitat the community conservation area provides in terms of, the availability of sufficient food throughout the year, a low number of predators, and relatively higher reproductive success (they care more for their offspring and produce offspring at any season) could make them among the most abundant. The highest frequency of sighting *Phacochoerus* *africanus* and *Papio* *anubis* could be attributed to the improved floristic diversity and structure, which in turn favored improved food, cover, and water sources for the large mammal species [24].

Colobus monkeys prefer trees available in the DEGAMF and Plantation forest of the study area. They have low risk of being ambushed by predators because most of their time is spent on tree branches to forage. They also have the same habitat preference; sufficient food throughout the year (different vegetation forage of leaves (mature and young leaves of plants), fruits or flowers, barks and shoots) and reproductive success (produce offspring at any season) prevail in the area, which could boost their abundance. Likewise, [20, 36] stated that the Colobus monkey prefers big trees that provide sufficient forage and cover opportunities. The abundance of tall evergreen characteristic tree species in the Dry Evergreen Afromontane Forest, such as *Juniperus* *procera*, *Podocarpus* *falcatus*, *Hagenia* *abyssinica*, *Maytenus* *addat*, and *Rapanea* *melanophloeos* creates favorite food and cover sources for the colobus monkey [22, 24]. Likewise, the improved forest cover due to the establishment of the community conservation area could create conducive habitat conditions through improved cover and food sources for the large mammal species.

The relatively higher frequency of sightings of the endemic and endangered *T*. *buxtoni* is probably attributed to the availability of both Dry Evergreen Afromontane forest and Sub-afro-alpine (Ericaceous scrubland) habitats that could produce sufficient food and cover for them. This study was comparable with the findings of [37–39], which stated that the distribution of Mountain nyala was distinct with different forest zones, from the ranges of 3000 to 4377 m and they preferred different habitat types. The distribution of *T*. *buxtoni* in available habitats is often influenced by several factors, such as habitat quality and suitability [37, 38].

### Population structure

During the study period, a higher number of adult individuals were counted and sighted than sub-adults and young ones. Among the populations of *Tragelaphus* *buxtoni* and *Colobus* *guereza* counted and sighted in the community conservation area, female adults on average constituted almost double the number of male adults. This could be attributed to the fact that the male adults (bulls) of *Tragelaphus* *buxtoni* were allowed for trophy hunting, legal hunting that targets the harvesting of adult males of the species using a per-determined population census. The proportional ratio of male to female of 1:2 and 1:3 is acceptable for quota setting and could show the sustainability of population growth [38].

Many of the observed large mammal species had large proportions of adults (Male adults and female adults), especially female adults. This could indicate progressive or increased population growth in the future, more likely attributed to less availability of predators, good quality food, and minimal poaching activities. It has been indicated that an increase in the female population is always an indication of positive growth and sustainability of the species in the future or shows a healthy population [25].

There are also considerably high young populations, mostly among primate species, including some ungulates such as warthogs, indicating future population growth. Nearly for the past 2 decades, the area has been managed by the community, whereby the local community is responsible for controlling any threats to the conservation of mammals and their habitats and they benefit from ecotourism revenues (important eco-tourism sites), trophy hunting, and the sale of managed forest products. The local communities in and around WAJIB benefit from trophy hunting fees and ecotourism activities such as viewing both mammals and birds, mountain trekking, and researchers' fees [40]. Additionally, they got 60% of the income generated from the sale of timber plantations from 2018 to 2019) i.e., the community gets 60% from all activities, including trophy hunting concessions [41, 42]. Currently, Trophy hunting in Dodola produces a substantial amount of money for the regional economy and 60% for local communities around the conservation area. This could create a sense of ownership and encourage the local community to devote themselves to wildlife conservation activities. This could be taken as an indicator of the success of the community conservation area for sustainable wildlife management.

## Conclusion and recommendations

The present study revealed that Dodola Community Conservation Area is home to diverse large mammalian species, including the endemic and vulnerable *Chlorocebus* *djamdjamensis*, endangered and endemic *Tragelaphus* *buxtoni*, endemic and critically endangered *Canis* *simensis* and the endemic subspecies *Tragelaphus* *sriptus* *menelik.* This makes it comparable with national parks, including the adjacent BMNP, which is a world biodiversity hotspot site.

There could be high population gains among most primate species and some ungulates like the warthog and the endangered and endemic mountain nyala. On top of that, the community conservation area can serve as movement corridors and alternative habitats for the large mammals in the adjacent BMNP. All these signify the importance of the community conservation area for sustainable management.

Despite the observed potential of Dodola Community Conservation Area to support high species richness and a large mammal community, many challenges are threatening the long-term integrity of the community conservation area, including the rapidly growing human population, expansion of agriculture and human settlement, high frequency of livestock grazing in areas designated for wildlife, poaching, tree cutting, human-wildlife conflicts, and power struggles among WAJIB stakeholders. Therefore, we recommend the participatory approach be strengthened to ensure sustainable co-existence between people and wildlife, with due emphasis on alleviating those conservation problems that otherwise jeopardize the effectiveness of the participatory conservation approach.

## Materials and methods

### Study area

**Fig. 2:**
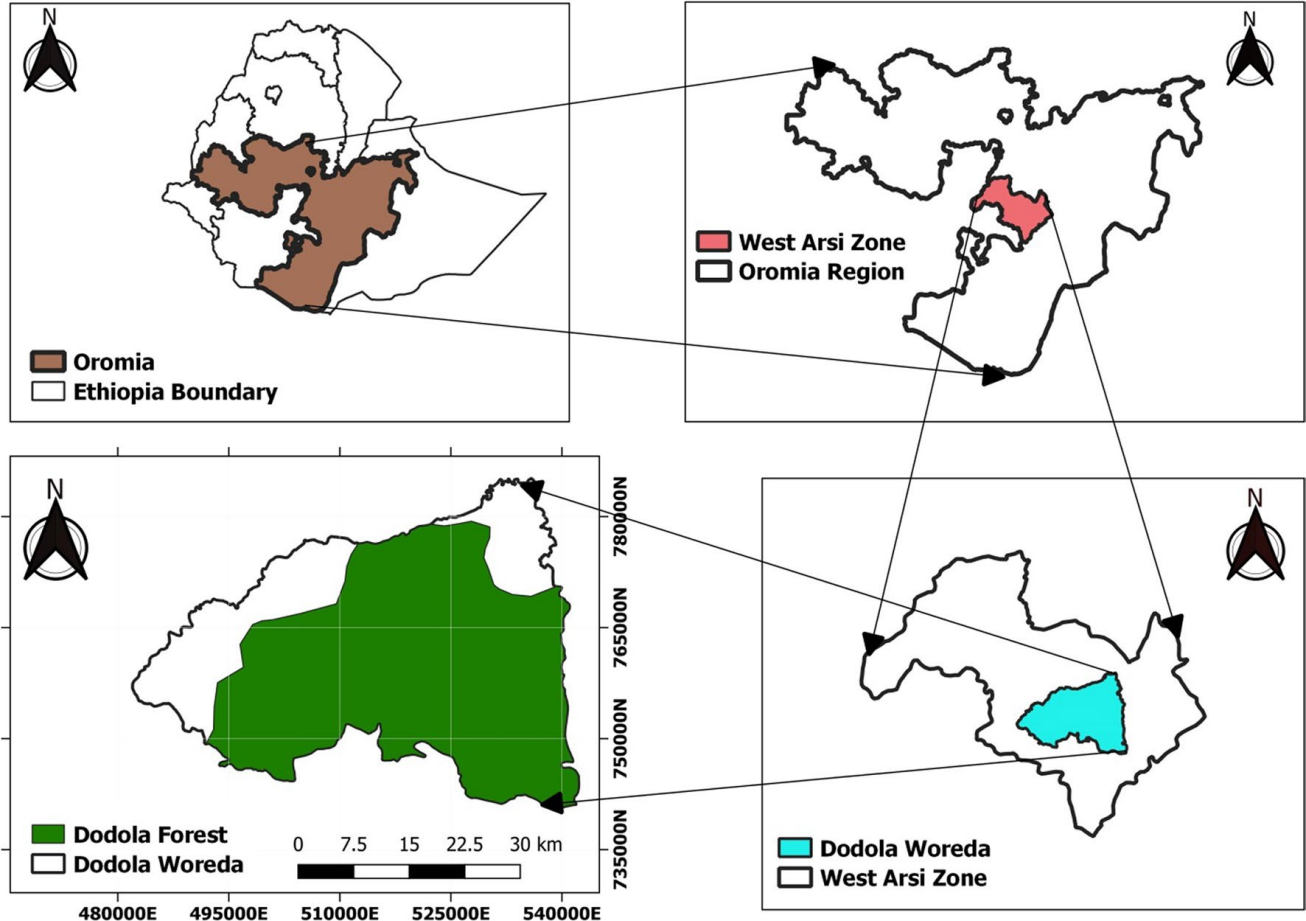
Location map of the study area

Dodola Community Forest is located in Southeastern Ethiopia in the Oromia Regional State of the West Arsi Zone in the Dodola district. It is located between 735,000 UTM N-780000 UTM N latitudes and 480,000 UTM E-540000 UTM E longitudes (Fig. 2). It is about 325 km from Addis Ababa (the capital city of Ethiopia) and 70 km from Shashemane town. The altitudinal range of the forest is from 2400 to 3712 m a.s.l. The daily temperature varies between 9 and 26 °C and the annual rainfall varies between 805 and 1260 mm [22].

Three vegetation types represent the Dodola community conservation area namely; Dry Evergreen Afro-montane forest (in the middle and lower altitudes and it ranges between 2400 and 3200 m a.s.l), Sub-afro-alpine habitat (Ericaceous scrubland vegetation from 3200 to 3714 m a.s.l) at the top altitudes and plantation forest (at the lower altitudes, from 2510 to 2875 m a.s.l) [22, 23]. The dominant tree species in the Dry Evergreen Afro-montane forest habitat are; *Juniperus* *procera*, *Podocarpus* *falcatus*, *Hagenia* *abyssinica*, *Maytenus* *addat*, *Hypericum* *lanceolatum*, and *Rapanea* *melanophloeos*, which are intermixed with Ericaceous vegetation at the upper belt of the dry evergreen Afro-montane forest [22, 23]. The Sub-afro-alpine habitat is dominated by *Erica* *arborea* and *Erica* *tremeria*. The plantation forest is dominated by *Cupressus* *lusitanica* and *Eucalyptus* *globules* [22].

Dodola Community Conservation area was established and implemented for the first time in 2000 by GTZ, the German Technical Development Organization. The community conservation system is locally known as WAJIB (an abbreviation in the ‘Afan Oromo’ language for forest dwellers association, or in Afan Oromo, ‘Waldaa Jiraattota Bosonaa’). The local communities in and around WAJIB benefit from trophy hunting fees and ecotourism activities such as viewing both mammals and birds, mountain trekking, and researchers' fees [41]. Additionally, from 2018 to 2019, they got 60% of the income generated from the sale of wood from state owned plantations) i.e., the community gets 60% from all activities, including trophy hunting [41]. Currently, 60% of the Trophy hunting revenue of large mammals in the Dodola Community Conservation Area is also shared with local communities around the conservation area [42]. But, the sustainability of these hunting areas is highly determined by the availability of viable populations in the forest area and the support of the local people. The large mammals of the Dodola forest area also generate income from visual tourism through entrance fees, assistance (guiding), renting horses (horse providers), and hut keepers (providing accommodation to tourists) [42].

### Sampling design

With the help of GIS and remote sensing technologies, the study area (45,683.29 ha) was stratified as Dry Evergreen Afromontane Forest (DEAMF) (25,102.9 ha), Sub-afro-alpine (Ericaceous Habitat) (20,055 ha) and Plantation Forest (525.39 ha). A total area of ten percent (4568.33 ha) of the study area was covered and sampled. Accordingly, proportional to the size of the study area, the sampled area was distributed as follows: 2510.29 ha out of 25,102.9 DEAMF ha, 2005.5 ha, out of 20,055 ha of Erica scrubland, and 52.5 ha out of 525.39 ha of plantation forest.

A total of 49 line transects were systematically established from the three major habitat types: 20 in the Erica scrub land habitat (Sub-afro-alpine), 27 in the Dry Evergreen Afro-montane forest and 2 (T48-T49) in the Plantation forest, to census large mammal abundance and distribution. The length and width of the transect line varied from habitat to habitat because of the topography, vegetation structure, and diversity; the length of the line transect in the sub-afro-alpine (Erica scrubland habitat) was 2.5 km and the maximum sighting distance was 300 m, in Dry Evergreen Afro-montane forest transect line length of 1.5 km and 200 m width was used, while in the plantation forest the transect line length was 2 km and 200 m width was used. The distance between two adjacent transects ranged from 1 to 1.5 km to avoid double counting of the same individuals and transects were transversed against the direction of the wind to minimize disturbance [19, 43, 44].

### Data collection

Data collection was carried out for both dry and wet seasons from December to July 2021. Large wild mammal identification and recording were carried out by direct observation aided with binoculars. Data collection was carried out by walking on foot with a constant speed of 1 km per hour along each transect and directly counting all individuals of every species sighted using the naked eye and 7 × 50 mm binoculars. When an individual animal or group of animals (every individual) was sighted, walking stopped, species name, individual numbers, age, sex, group size, perpendicular distance of observation guessed, GPS location, weather, the activity of animals, and vegetation types were recorded on the pre-prepared data sheet [26, 29, 30]. To avoid double counting of the same individual, individuals seen within a distance of < 50 m from the nearby group were recorded as members of the same group.

Morphological development (body size, horn ridges, and size), growth and maturation, changes in shapes (pelage color or patterns), and sexual maturity (condition of mammary glands, behavior during breeding) were used to determine the approximate age; adult, sub-adult and young [25]. The sexual characteristics, external genitalia and behavior (urination posture, vocalizations, nipples, descended tests), and sexually dimorphic characteristics (such as the absence or presence of horns) were used to determine the sex. Photographs were also taken for further confirmation.

Surveys of large wild mammals were carried out during early morning hours (6:00 to 10:00 a.m.) and late afternoon hours (3:00 to 5:00 p.m.), when most mammals were more active [17, 19, 26, 30]. Sampling was done for a total of forty days, twenty days in each wet and dry season. The total transect walked over the study period was 4000 km, 3240 km and 329 km in the Erica, DEAMF and plantation habitats, respectively. Trained field assistants were employed to survey transects located in a similar topographic landscape at the same time to minimize the movements of animals between transects, thereby avoiding double-counting.

There are certain situations in which none of the direct methods can be applied to secretive and nocturnal mammals. Therefore, to supplement the direct observation, all fresh scats of the wild large mammals were recorded. The identification of scats obtained was attempted in the field by using specialized field guides for the identification of scats of mammals.

### Data analysis

Sorenson's Index (SI) was used to calculate species similarity between each habitat. The Sorenson's Index of species similarity among the habitat types was computed using the formula below: SI = 2C/S_1_ + S_2_; where C is the number of species the two habitats have in common, S_1_ is the total number of species found in habitat 1, and S_2_ is the total number of species found in habitat 2. The frequency of sightings per sampling effort/per given km of distance walked was computed using Microsoft Excel 2010. The frequency of sightings of a particular large mammal species is the number of individuals observed and counted during the survey per given km of distance covered/walked.

## Data Availability

The data that support the research findings will be made available up on request by the corresponding author.

## Abbreviations

a.s.l: Above sea level
BMNP: Bale Mountains National Park
Kebeles: (AT-Aloshe Tilo, WT-Waltai Tosha, WA-Waltai Azira, FA-Fassil Angesso, IS-Ititu Sura)
AMNP: Arsi Mountains National Park
BMN: Bale Mountains National Park
DEAMF: Dry Evergreen Afromontane forest
OFWE: Oromia Forest and Wildlife Enterprise
PWM: Participatory Wildlife Conservation
WAJIB: Is an abbreviation in the ‘Afan Oromo’ language for forest dwellers association or in Afan Oromo, ‘Waldaa Jiraattota Bosonaa’
